# Evaluating the Toxicity Induced by Bisphenol F in *Labeo rohita* Fish Using Multiple Biomarker Approach

**DOI:** 10.1155/2024/8646751

**Published:** 2024-11-08

**Authors:** Shabbir Ahmad, Hasnain Akmal, Khurram Shahzad, Muhammad Khalil Ahmad Khan, Farhat Jabeen

**Affiliations:** ^1^Department of Zoology, University of Okara, Okara 56130, Pakistan; ^2^Department of Zoology, Government College University Faisalabad, Faisalabad 37251, Pakistan

**Keywords:** aquatic toxicology, biomarkers, emerging pollutants, genotoxicity, histology, immunotoxicity

## Abstract

Bisphenol F (BPF) is an emerging contaminant extensively used in the pharmaceutical, chemical, and food industries, exerting deleterious effects on human and wildlife health. Therefore, the current study was conducted to assess the toxicity induced by BPF in rohu *Labeo rohita* using multiple biomarkers such as oxidative stress, activity of antioxidant enzymes, biochemical parameters, histology, and genotoxicity. Fish were separated into four groups (T1–T4). Group T1 served as a control (0 μg/L), while Groups T2, T3, and T4 were exposed to BPF concentrations of 600 μg/L, 1200 μg/L, and 1800 μg/L, respectively, for 21 days. Results showed a significant (*p* < 0.05) increase in oxidative biomarkers (thiobarbituric acid reactive substance [TBARS] and reactive oxygen species [ROS]), while the concentration of antioxidant biomarkers (peroxidase [POD], superoxide dismutase [SOD], reduced glutathione [GSH], and catalase) was significantly (*p* < 0.05) decreased with the rising concentration of BPF in the liver, gills, and kidney of fish. Significant reduction (*p* < 0.05) in biochemical parameters was measured from collected whole blood, including red blood cells (RBCs), hemoglobin (HGB), mean corpuscular HGB (MCH), MC volume (MCV), hematocrit (HCT), MC HGB concentration (MCHC), platelets, low-density lipoprotein (LDL), cholesterol, high-density lipoprotein (HDL), total proteins, very LDL (VLDL), albumin and globulin, while white blood cells (WBCs), neutrophils, triglycerides, aspartate aminotransferase (AST), blood glucose, and alanine transaminase (ALT) levels were increased significantly (*p* < 0.05). Comet assay showed the DNA damage potential of BPF in erythrocytes. Histological examination showed that exposure to BPF causes several degenerative effects in the soft tissues (gills, liver, and kidney) of treated fish. It is concluded that BPF induces deleterious effects via disruptions in histological, genotoxic, and biochemical alterations in several organs of exposed fish.

## 1. Introduction

Bisphenol F (BPF) (BPA analog) is a raw chemical commonly used in pharmaceutical, chemical, and food production [1]. The most common BP is BPA, which causes many human diseases, such as cognitive impairments and neurodevelopmental disorders in newborns and young children. It causes toxicity and abnormalities in humans and wildlife [2, 3]. Due to its highly toxic nature, BPA has been banned since 2010 [4]. Simultaneously, BPF, a significant alternative to BPA, has been used in industries. BPF is used in dental sealants, food packaging, grouts, electrical varnishes, pipe linings, plastics, industrial floors, adhesives, and lacquers [5]. The occurrence of BPF in the environment is increasing with its use, as BPF concentration increased in Taihu Lake (China), ranging from 0.83 ng/L to 723 ng/L in 5 years [6, 7]. Due to its better resistance and low viscosity to solvents compared to BPA, it is finding increased usage [8]. It continuously accumulates in terrestrial and aquatic animals due to environmental contamination. BPF enters the human body by ingesting or exposure to surrounding environmental media [9]. The level of BPF in human blood was three times greater than that of BPA [10]. In the United States, BPF was detected in 66.5% of urine collected from children and adults [11]. It was also found in cord plasma, breast milk, maternal blood [12], white matter, and the hypothalamus [13]. Ji-Youn et al. [14] reported that BPF poses a similar cancer risk as BPA. Usman, Ikhlas, and Ahmad [15] reported that BPF can damage DNA in HepG2, chicken DT40, and MCF-7 cell lines and cause oxidative damage to DNA bases.

BPF was detected in several environments, such as soil, indoor dust, and water [16]. BPF enters the water bodies through industrial wastes and is deposited in the water and sediments. In recent years, due to widespread industrial usage, the level of BPF has significantly increased in water bodies [5]. BPF and other suspended particles can easily reach natural water bodies [17]. It was found in natural water bodies and multiple drinking water sources, with levels ranging from tens to hundreds μg/L [6, 18]. BPF was the most commonly detected pollutant, and the BPF concentration was one to two times greater than BPA in water samples collected from rivers and oceans in some Asian countries. BPF was detected in freshwater bodies of China (Luoma Lake 3.5–14 ng/L [16], Pearl River 448–1110 ng/L), Korea (Han River 121–1300 ng/L), and Japan (Tokyo Bay 90–2850 ng/L) [19]. So the adverse effects of BPF on aquatic animals have increasingly attracted attention [19]. Dos Santos et al. [20] reported that BPF (500 μg/L) causes various physiological and behavioral effects in zebrafish, such as developmental defects, immunotoxicity, endocrine disruption, neurotoxicity, and oxidative stress. Studies have shown that BPF (50 μg/L–1 mg/L) has estrogenic, genotoxic, neurotoxic, and cytotoxic effects on some aquatic species that are comparable to or more severe than those of BPA [21–24]. It is developmentally hazardous in Chinese Medaka and produces endocrine-related abnormalities in the gene transcriptional network [25]. It also causes oxidative testicular impairment in rats [26]. In zebrafish, BPF causes oxidative stress, decreased locomotor behavior, and delayed neurological development [27]. It also causes immunotoxicity, oxidative stress, and metabolic detoxification disruption in three-spined stickleback fish [28]. So, studying the toxicity and possible adverse effects of BPF on fish and other aquatic animals is very important.

The literature about the adverse effects of BPF on aquatic animals, especially fish, is scant. Specifically, the toxic effects of BPF on biochemical biomarkers, DNA damage, and histological alterations in freshwater fish are unclear. Therefore, the current study was conducted to assess the toxic effects of BPF on oxidative and antioxidant biomarkers and biochemical, histological, and genotoxic variations in rohu *Labeo rohita*. Nevertheless, BPF may induce toxicity to aquatic organisms, especially fish.

## 2. Materials and Methods

### 2.1. Experimental Animal

The current trial was conducted in the fish lab of the Department of Fisheries, University of Okara, Pakistan. In this research, all actions were carried out on fish by following the Ethical Committee of the University of Okara, Punjab, Pakistan. Healthy *L. rohita* (*n* = 90, 50% male and 50% female, aged two months, average weight 34.12 ± 2.24 g, and length 17.71 ± 0.43 cm) were obtained from a Manawa Fish Hatchery, Lahore, Pakistan and safely transported to the aquaculture lab of department of fisheries of University of Okara, Pakistan, and stocked in a large glass aquarium having dimensions 40ʺ *L* × 45ʺ *W* × 35ʺ *H* with 100 L water. The fish were rinsed with a 0.1% KMnO_4_ solution and acclimatized in glass aquariums under standard lab conditions at 27.0 ± 0.12°C temperature for 7 days. Fish were fed daily with commercially formulated feed (22% protein). During transportation and acclimatization, no mortality was observed.

### 2.2. Chemicals

BPF 98% purity (CAS: 620-92-8; catalog: B838403) was obtained from MACKLIN, China. An appropriate amount of BPF (1g) was dissolved in ethanol 99.99% purity (1000 mL) to make a stock solution. Clove oil was purchased from Al- Fatah herbal store, Pakistan.

### 2.3. Experiment Design

After acclimatization, three glass aquaria were designated to each of four treatment groups (T1, T2, T3, and T4), and each aquarium had a total number of 07 fish. The T1 group was considered a control, while groups T2, T3, and T4 were exposed to three concentrations of BPF such as 600, 1200, and 1800 μg/L, respectively, for 21 days. Concentrations of BPF were made based on the 96 h LC_50_ value. The 96 h LC_50_ value for BPF in *L. rohita* was 5500 μg/L (5.50 mg/L). Each aquarium was aerated with an air pump. 90% of the water from each aquarium was replaced with freshwater daily to remove waste. During the whole experiment period, water physicochemical properties were measured as temperature 27.0 ± 0.17°C, pH 8.93 ± 0.17, DO 8.09 ± 0.14 mg/L, total dissolved solid 18.21 ± 3.02 mg/L, total hardness 17.13 ± 0.13 mg/L, chlorides 7.3 ± 0.02 mg/L, magnesium 13.9 ± 0.21 mg/L, sodium 12.6 ± 0.16 mg/L, potassium 1.6 ± 0.09 mg/L, and calcium 37.3 ± 0.13 mg/L.

### 2.4. Biochemical Analysis

On Day 21, all fish were removed from all groups. Clove oil (0.4 mL/L) was mixed with a little warm water to euthanize fish by following the method of Davis et al. [29]. Blood was collected from the caudal vein via BD syringe and stored in EDTA vials for hematology and gel vials for serology. Then, it was moved to the hematology lab and analyzed at standard storage conditions and room temperature. Various hematological indices, including red blood cells (RBCs), hemoglobin (HGB), mean corpuscular HGB (MCH), MC volume (MCV), hematocrit (HCT), MC HGB concentration (MCHC), white blood cells (WBCs), platelets (PLT), and neutrophils were analyzed by the hematological analyzer (SMT-50 Hematology Analyzer) under laboratory conditions.

For serology, samples were left to coagulate over 15 minutes. The serum was separated from blood samples by centrifuging them for 10 min at 5000 rpm, then stored at −20°C for further biochemical analysis. Concentrations of serum AST, ALT, blood glucose, lipid profile, and serum proteins were determined using assay kits provided by Diamond Diagnostic, Egypt. A spectrophotometer (Microlab 200, UVG 060609) was used to analyze the samples.

### 2.5. Comet Assay

The comet analysis was carried out using a three-step process (lysis, electrophoresis, and staining) to assess DNA damage in erythrocytes under alkaline conditions following the method of Singh et al. [30]. After blood collection, blood (20 μL) was diluted in phosphate-saline buffer (980 μL). One percent (w/v) normal melting point agarose was incubated for 24 h at 37°C to dry the layer. A second layer was applied, which consisted of diluted blood (20 μL) and low melting-point agarose (80 μL). A cover slip was placed over the slides and left at 4°C for 10 min. Slides were placed in the electrophoretic buffer for 20 min. For the preparation of electrophoresis buffer, NaOH solution (18 mL) and EDTA (ethylene diamine tetra acetic acid) solution (3 mL) were mixed with double-distilled water. A final volume of 1000 mL was prepared at pH 13. Electrophoresis was performed for 20 min at 0.55 V/cm and 300 mA. After that, the slides were neutralized for 15 min using the neutralization buffer and kept overnight to dry. Afterward, slides were stained using an ethidium bromide solution. A stock solution was prepared by adding ethidium bromide (10 g) in double-distilled water (50 mL). For the preparation of the working solution, stock solution (1 mL) was mixed with double-distilled water (9 mL). After this, slides were observed under a fluorescent microscope at 400×. Comet IV computer software was used to score microscopic photographs of comets following the method described by Chaubey [31].

### 2.6. Oxidative Stress and Antioxidant Estimation

After blood collection, fish (*n* = 3 for each group) were dissected humanely, and organs (gills, liver, and kidney) were collected. For oxidative and antioxidant biomarkers, an automated homogenizer was used to homogenize the tissues in a phosphate buffer saline solution and centrifuge them for 30 min at 30,000 rpm. After that, the supernatant was collected in Eppendorf and stored at −20°C until further analysis. The oxidative and antioxidant biomarkers were estimated using commercially available diagnostic reagent kits according to the instructions (ELISA Kits Manufacturer, United States of America). Reactive oxygen species (ROS) content was assessed following the method of Hayashi et al. [32]. TBARS and GSH were estimated in the homogenate following the procedure described by Iqbal et al. [33]. The catalase activity (CAT) was estimated in the homogenate following the method of Afsar and Razak [34]. Activity of superoxide dismutase (SOD) was assessed by the method described by Afsar et al. [35]. Peroxidase (POD) activity in homogenate was assessed following the protocol of Chance and Maehly [36].

### 2.7. Histology

For histology, tissue samples were preserved in a 10% formalin aqueous solution at room temperature immediately after dissection for 24 h. After that, tissue samples were dehydrated (using a graded series of ethanol/water washes), diaphonized, and embedded in paraffin. Then, an automated microtome (Leica RM-2155) was used to cut sections 5 µm thick. Eosin and hematoxylin were used to stain the slides according to the procedure described by Silva et al. [37]. Stained slides were visualized at 40× with an optical microscope (Leica DM2500, Japan).

### 2.8. Statistical Analysis

The measured data are shown as mean ± S.D in this research. All of the data that had been compiled followed a normal distribution pattern. All statistical analyses and graphic design were undertaken using Graph Prism Pad software (version 9.5.1). For genotoxic examination (olive tail moment [OTM] and % of tail DNA damage), microscopic photographs of the comet were scored by Comet IV computer software. The significant difference in means (mean ± S.D) of hematobiochemical, oxidative, and antioxidant biomarkers, OTM, and % of tail DNA damage of control and experimental groups was analyzed using a one-way analysis of variance (ANOVA) followed by a Dunnett post hoc comparison test. The statistical significance levels were determined to be ⁣^∗∗∗^*p* < 0.001, ⁣^∗∗^*p* < 0.01, and ⁣^∗^*p* < 0.05. Stained slides were examined under an optical microscope (Leica DM2500, Japan) at 40× for histological examination.

## 3. Results

### 3.1. Biochemical Analysis

Biochemical results are shown in Table 1. According to the hematological analysis, a significant reduction was observed in RBCs, HGB, MCH, MCV, HCT, MCHC, and platelet contents of BPF-exposed fish compared to the control group. WBCs and neutrophils were found to be increased. The results of the biochemical analysis showed a significant increase in triglycerides, AST, ALT, and blood glucose levels. In contrast, lipid profile (cholesterol, LDL, HDL, and VLDL) and serum proteins (albumin, total proteins, and globulin) levels were significantly decreased in BPF-exposed fish.

### 3.2. Oxidative and Antioxidant Biomarkers' Observations

**Figure 1:**
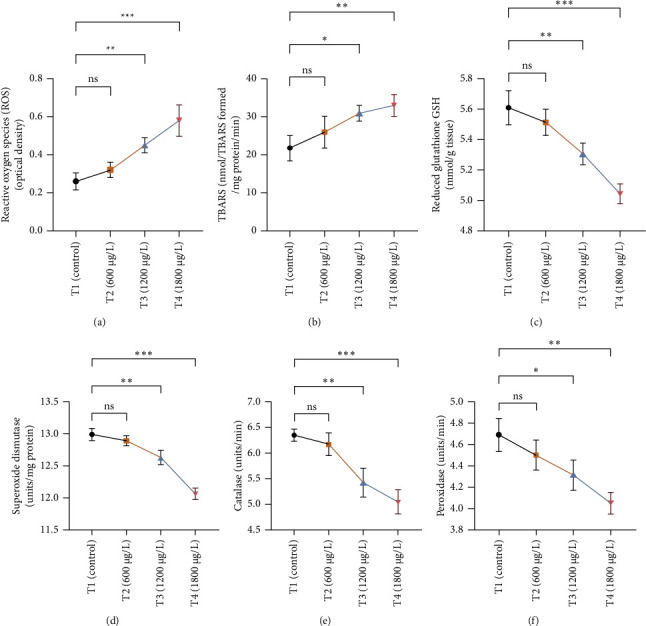
The variations in the levels of (a) reactive oxygen species (ROS), (b) thiobarbituric acid reactive substance (TBARS), (c) reduced glutathione (GSH), (d) superoxidase dismutase, (e) catalase, and (f) peroxidase in the supernatant of the liver of *L. rohita* between T1 (control) and three (T2, T3, and T4) BPF-treated groups. The data are represented as mean ± SD. Asterisks represent significant differences between T1 (control) and three (T2, T3, and T4) BPF-treated groups (analyzed by one-way ANOVA by applying Dunnett's post hoc comparison test; ⁣^∗^*p* < 0.05, ⁣^∗∗^*p* < 0.01, and ⁣^∗∗∗^*p* < 0.001). *n* = 3 for each experimental group. ns = nonsignificant.

**Figure 2:**
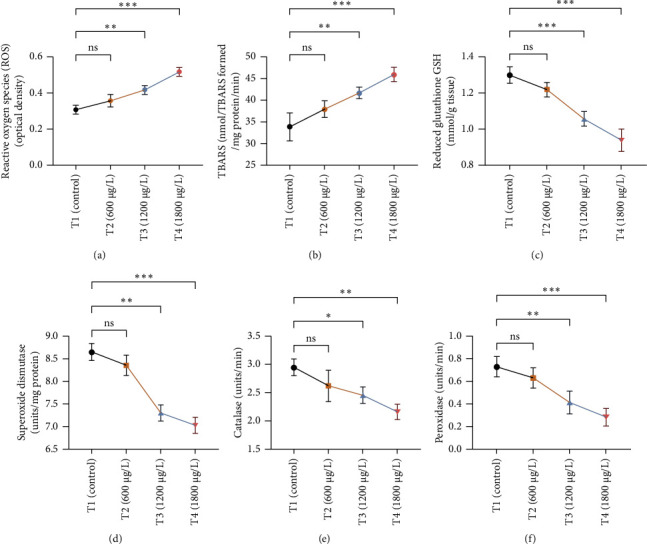
The variations in the levels of (a) reactive oxygen species (ROS), (b) thiobarbituric acid reactive substance (TBARS), (c) reduced glutathione (GSH), (d) superoxidase dismutase, (e) catalase, and (f) peroxidase in the supernatant of the gills of *L. rohita* between T1 (control) and three (T2, T3, and T4) BPF-treated groups. The data are represented as mean ± SD. Asterisks represent significant differences between T1 (control) and three (T2, T3, and T4) BPF-treated groups (analyzed by one-way ANOVA by applying Dunnett's post hoc comparison test; ⁣^∗^*p* < 0.05,⁣^∗∗^*p* < 0.01, and ⁣^∗∗∗^*p* < 0.001). *n* = 3 for each experimental group. ns = nonsignificant.

**Figure 3:**
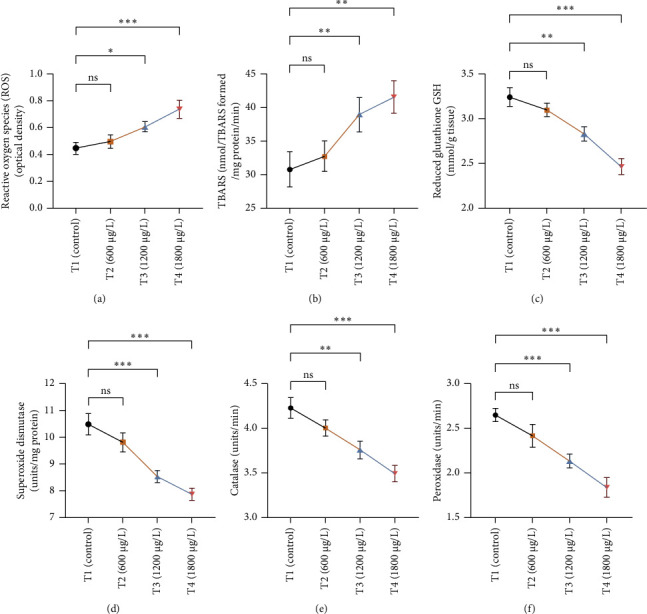
The variations in the levels of (a) reactive oxygen species (ROS), (b) thiobarbituric acid reactive substance (TBARS), (c) reduced glutathione (GSH), (d) superoxidase dismutase, (e) catalase, and (f) peroxidase in the supernatant of the kidney of *L. rohita* between T1 (control) and three (T2, T3, and T4) BPF-treated groups. The data are represented as mean ± SD. Asterisks represent significant differences between T1 (control) and three (T2, T3, and T4) BPF-treated groups (analyzed by one-way ANOVA by applying Dunnett's post hoc comparison test; ⁣^∗^*p* < 0.05, ⁣^∗∗^*p* < 0.01, and ⁣^∗∗∗^*p* < 0.001). *n* = 3 for each experimental group. ns = nonsignificant.

The results of oxidative and antioxidant biomarkers in the kidneys, liver, and gills of *L. rohita* are presented in Figures 1, 2, 3. According to the current results, TBARS and ROS contents were significantly increased in the soft tissues of the liver of exposed fish (1200 μg/L and 1800 μg/L), while no significant increase was observed in TBARS and ROS contents in soft tissues of the liver of exposed fish (600 μg/L) as compared with unexposed fish. Antioxidant biomarker enzymes such as catalase, POD, GSH, and superoxidase dismutase significantly decreased in soft tissues of the liver of exposed fish (1200 μg/L and 1800 μg/L), while no significant decrease was observed in antioxidant biomarker enzymes in soft tissues of the liver of exposed fish (600 μg/L) as compared with unexposed fish (Figure 1). TBARS and ROS contents were significantly increased in the soft tissues of the gills of exposed fish (1200 μg/L and 1800 μg/L), while no significant increase was observed in TBARS and ROS contents in the soft tissues of gills of exposed fish (600 μg/L) as compared with unexposed fish. Antioxidant biomarkers such as catalase, POD, GSH, and superoxidase dismutase significantly decreased in soft tissues of the gills of exposed fish (1200 μg/L and 1800 μg/L), while no significant decrease was observed in antioxidant biomarkers in soft tissues of the gills of exposed fish (600 μg/L) as compared with unexposed fish (Figure 2). The content of TBARS and ROS increased significantly in the soft tissues of the kidneys of fish if exposed to 1200 μg/L and 1800 μg/L quantities of BPF. No significant change was observed between unexposed and BPF-exposed fish (600 μg/L). Antioxidant biomarkers (catalase, superoxidase dismutase, GSH, and POD) were significantly reduced in the soft tissues of the kidneys of fish treated with 1200 μg/L and 1800 μg/L of BPF. However, no significant change was observed between unexposed fish and exposed fish (600 μg/L) as shown in Figure 3.

### 3.3. Genotoxic Examination

**Figure 4:**
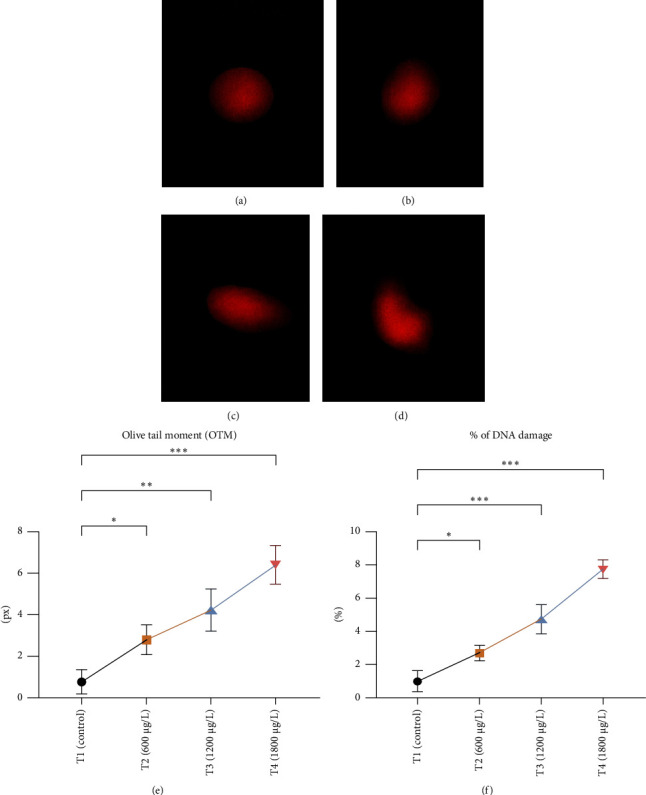
DNA damage in erythrocytes observed by comet assay. Photographs of comet assay show (a) normal erythrocyte cells; (b–d) show nuclear material damage observed under a fluorescent microscope at 400× and scored microscopic photographs of the comet showing (e) olive tail moment and (f) % of DNA damage by using Comet IV computer software. The data are represented as mean ± SD. Asterisks represent significant differences between T1 (control) and three (T2, T3, and T4) BPF-treated groups (analyzed by one-way ANOVA by applying Dunnett's post hoc comparison test; ⁣^∗^*p* < 0.05,⁣^∗∗^*p* < 0.01, and ⁣^∗∗∗^*p* < 0.001). *n* = 3 for each experimental group. ns = nonsignificant.

The DNA damages were measured as OTM (DNA distribution in the tail) and percentage DNA in the blood cells of the control and exposed fish using a comet assay. The DNA strand breaks after exposure to BPF in *L. rohita*, as shown in Figure 4. A significant increase was observed in the DNA damage percentage at a high BPF dose (1800 μg/L). A significant difference was observed throughout the increase in BPF doses from T1 (0 μg/L), T2 (600 μg/L), T3 (1200 μg/L), and T4 (1800 μg/L). A dose-dependent increase was observed among T1 (control) and three exposed groups in the OTM.

### 3.4. Histological Examination

**Figure 5:**
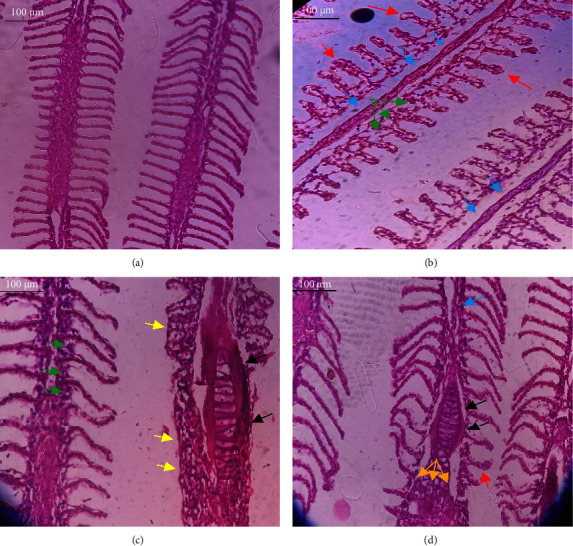
Microphotographs of gill tissues of *L. rohita* show (a) normal arrangement of gill filaments including primary and secondary gill lamellae of control fish; (b–d) show various histological alterations such as edema (black arrow), blood cognition (red arrow), fusion and degeneration of primary lamellae (yellow arrow), bone cell deformities (blue arrow), hyperplasia of epithelial cells (green arrow), and necrotic cells (orange arrow) in the gills of *L. rohita* exposed to 600, 1200, and 1800 μg/L BPF, respectively. Slides were visualized at 40×. Bar = 100 μm with an optical microscope (Leica DM2500, Japan).

**Figure 6:**
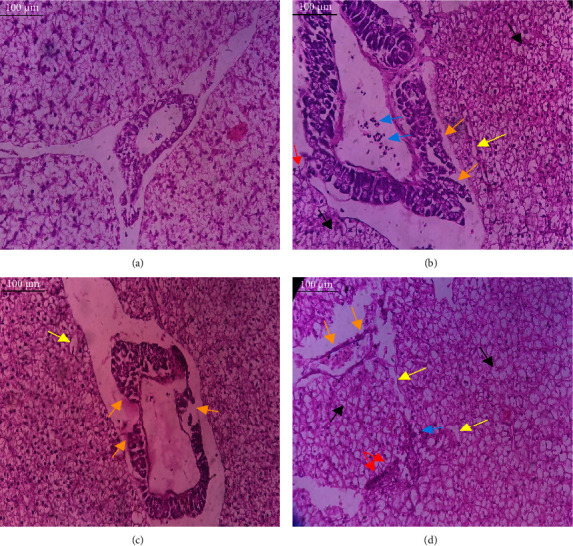
Microphotographs of liver tissues of *L. rohita* show (a) normal arrangement of hepatic cells of control fish; (b–d) show various histological alterations such as degenerative nuclei (black arrow), necrotic cells (red arrow), damaged central vein (orange arrow), sinusoidal spaces (yellow arrow), and formation of cluster nuclei (blue arrow) in the liver of *L. rohita* exposed to 600, 1200, and 1800 μg/L BPF, respectively. Slides were visualized at 40×. Bar = 100 μm with an optical microscope (Leica DM2500, Japan).

**Figure 7:**
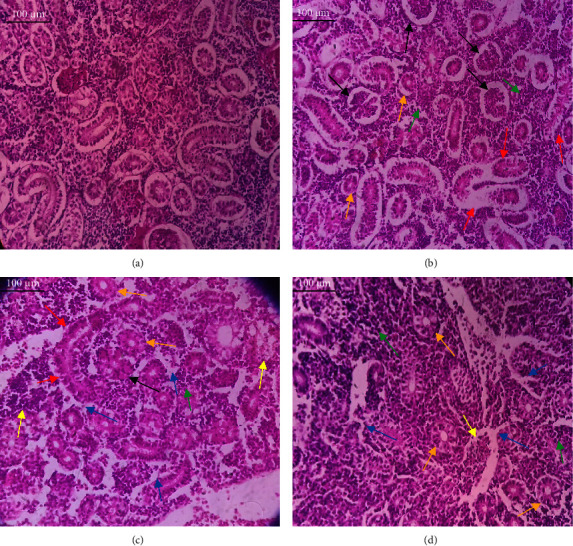
Microphotographs of kidney tissues of *L. rohita* show (a) normal arrangement of kidney cells and renal tubules of control fish; (b–d) show various histological alterations such as melanomacrophage (black arrow), elongated tubules (red arrow), tubules with hypertrophied nuclei (orange arrow), sinusoidal spaces (blue arrow), formation of cluster nuclei (green arrow), and necrotic cells (yellow arrow) in the kidney of *L. rohita* exposed to 600, 1200, and 1800 μg/L BPF, respectively. Slides were visualized at 40×. Bar = 100 μm with an optical microscope (Leica DM2500, Japan).

In the current study, *L. rohita*, exposed to different concentrations of BPF, showed various alterations in various organs, such as the liver, kidneys, and gills (Figures 5, 6, 7). Results of dose-dependent treatment showed moderate to severe histological alterations. Microphotographs of gill tissues of control fish showed no damage in gill filaments, including primary and secondary gill lamellae of control fish. Fish exposed to different doses of BPF showed various histological alterations such as edema, blood cognition, fusion and degeneration of primary lamellae, bone cell deformities, and hyperplasia of epithelial cells and necrotic cells in the gill tissues (Figure 5). No damage was observed in the hepatic cells of the control fish. BPF-exposed fish showed various histological alterations such as degenerative nuclei, necrotic cells, damaged central vein, sinusoidal spaces, and formation of cluster nuclei in liver tissues (Figure 6). Microphotographs of kidney tissues of control fish showed a normal arrangement of kidney cells and renal tubules. Various histological alterations such as melanomacrophage, elongated tubules, tubules with hypertrophied nuclei, sinusoidal spaces, and formation of cluster nuclei and necrotic cells in the kidney of fish exposed to three different doses of BPF (Figure 7).

## 4. Discussion

BPF is the most common BP in food products such as meat, fish, vegetables, and seafood [38]. It was expected that BPF would have the same environmental distribution as BPA due to chemical similarity [15]. Different organic and inorganic pollutants such as pesticides, heavy metals, and BPs may affect fish both directly and indirectly [24, 39]. Many environmental monitoring studies have reported that BPF poses a serious threat to humans and other animals, especially aquatic organisms, and will become a significant source of food contamination and environmental pollution in the future [40, 41]. The present in *vivo* research was conducted to investigate the adverse effects of BPF using *L. rohita* as a model organism through multiple biomarker approach.

Hematological variables are very important for evaluating pathological and physiological alterations in fish [42, 43]. Many studies have indicated that when contaminants alter water quality, the levels of one or more hematological parameters will reflect any physiological changes [44]. In the present study, some blood parameters such as HGB, MCH, RBCs, PLT, HCT, MCV, and MCHC levels were significantly decreased, and a significant rise in neutrophils and WBCs was observed in treated groups. To date, this is the first evidence that BPF induces hematological changes in rohu *L. rohita*. Higashihara et al. [45] reported that BPF (20–500 mg/kg/day for 28 days) causes a reduction in HGB, RBCs, and HCT levels in rats. Igarashi et al. [46] also reported that 4-benzylphenol (structural analog of BPF) causes a reduction in HGB, RBCs, HCT, MCV, MCH, and MCHC and PLT levels and elevation in WBCs and neutrophils levels in rats (30–750 mg/kg/day for 28 days). Similar findings were also reported by Afzal et al. [47] due to BPA in fish. Alterations in the shape and size of RBCs caused by environmental contaminants typically accelerate the removal of RBCs from the blood circulation [48]. BPF induced significant morphological alterations in RBCs [49]. BPF may reduce the iron level in the blood or lead to RBC degradation by changing the cell membrane's permeability, making RBCs more brittle and prone to hemolysis. The reduction of RBC may be associated with the elevation of free radicals or the reduction of antioxidant enzymes in the blood of BPF-exposed fish. This process causes lipid peroxidation in fish blood [50]. Reduction in HGB and HCT levels are logically attributed to RBC reduction in *L. rohita* due to BPF exposure. The MCHC serves as a valuable indicator for assessing RBC enlargement and a reduction in HGB production [51]. Generally, a decrease in these values often indicates anemia in fish and other animals [52]. Various animal stressors can increase WBCs due to immune system activation and inflammation [53]. A rise in the WBC level may be due to direct activation of immunological responses due to exposure to BPF or may be associated with tissue injury. The elevation in the count of WBCs in treated fish indicates a state of toxemia that indicates impairment of the defense system [54].

Serum biochemistry is an excellent biomarker for evaluating the adaptation ability, health, and dietary status of fish to their surroundings [55]. In the present research, high levels of triglycerides, AST, ALT, and blood glucose were observed in a dose-dependent manner, and cholesterol, LDL, HDL, VLDL, total proteins, globulin, and albumin levels were significantly decreased in BPF-exposed fish. This is the first experimental evidence that BPF induces changes in serum biochemical parameters in freshwater *L. rohita.* Higashihara et al. [45] reported that BPF causes a reduction in cholesterol, total proteins, albumin, and globulin concentrations in rats. Lee et al. [56] indicated that exposure to BPF causes a reduction in cholesterol, LDL, HDL, and VLDL levels in rats. Similar findings were documented earlier in fish due to BPA [57]. Changes in the liver caused by BPF exposure can increase oxidative damage that may increase AST and ALT levels [58]. BPF may disrupt glucose homeostasis in fish through inflammation, oxidative stress, β cell dysfunction, and insulin resistance [59]. Reduction in serum albumin, proteins, and globulin in BPF-treated fish may be because of kidney and liver dysfunctions [60]. A reduction in plasma total proteins might result from the breakdown of proteins to metabolically deal with stress as energetic substrates [61]. By disrupting oxidative processes, BPF may cause alterations in the lipid profile. Lipid abnormalities are a common symptom of diabetes mellitus. These variations may be further increased by increased environmental oxidization, which stimulates the formation of glycated LDLs, oxidized LDLs, and oxysterols. It suggested that these oxidized lipids may bind with activating inflammatory or specific receptor proteins, increasing ROS production [62].

Oxidative stress is a significant field of study in toxicology because oxidative damage and altered antioxidant levels may serve as early indicators of contamination [63]. Assessment of oxidative stress factors and antioxidant biomarkers (GSH, SOD, and CAT) are known as an excellent biomarker of immune responses and protect tissues from damage caused by free radicals and screen for adverse effects of pollutants [64, 65]. A balance was maintained in the production of ROS through cell metabolism and antioxidants in normal tissues, which is very important for the protection of oxidative damage. Disturbance in this balance may cause oxidative stress damage by increasing the production of ROS and decreasing the levels of antioxidants [66]. In the current investigation, oxidative stress indices (ROS and TBARS) were measured and found to be significantly increased in the kidneys, gills, and liver of exposed fish. No previous study has been reported about the oxidative damage in the kidneys, gills, and liver of rohu *L. rohita* due to BPF. Exposure to various organisms to different toxins causes quick and increased ROS production due to the detoxification processes of exposed organisms. High ROS production causes lipid peroxidation, which in turn causes abnormalities in the cellular membrane and TBARS production [67, 68]. Therefore, the higher content of oxidative stress in BPF-exposed fish in the current study may be due to misbalancing and depletion of antioxidant enzymes. Some in *vivo* investigations reported that BPF induced ROS production in marine rotifer (0.5–5 mg/L) [69], *Branchionus koreanus*, *Carpinus carpio* (0.1–1000 μg/L for two months) [70], zebrafish, and rats testes (5–50 μg/L for 48 weeks) [71]. Furthermore, higher oxidative stress may result from tissue damage, which may lead to an abnormal oxidative phosphorylation mechanism. Production of ROS primarily depends upon the cellular background, toxicant concentrations, duration, and exposure time [72]. Moreover, BPs induce oxidative damage by reducing various antioxidant enzymes in terrestrial and aquatic animals [64].

Current experimental study showed that BPF significantly reduced the levels of different antioxidant biomarkers (POD, GSH, SOD, and CAT) in the gills, kidneys, and liver of exposed *L. rohita*. This research is the first to provide evidence about the effects of BPF on various antioxidant biomarkers in different organs of rohu *L. rohita*. The decreased level of these enzymes in various fish tissues may be because of higher oxidative stress content and antioxidant enzyme depletion [68]. GSH is a vital component of the antioxidant defense mechanism in multiple tissues [73]. The GSH enzyme degrades lipid peroxides. Therefore, alterations in the activity of GSH may also be linked to increased lipid peroxidation [74]. In the previous literature, decreased antioxidant enzyme activities were reported in common carp [47] and *Hypophthalmichthys nobilis* [67] due to BPA (3.0–6 mg/L for 30 days). Reducing antioxidant biomarkers in several tissues might be associated with excessive free radical production due to BPF, leading to dysfunction and abnormalities in the antioxidant defense system [75]. Ghafarifarsani et al. [76] reported that different contaminants such as pesticides, heavy metals, and nanoparticles caused a reduction in the activity of antioxidant enzymes (SOD and CAT). It has been determined that the production of O_2_ radicals associated with oxidative stress oxidizes the enzyme's cysteine amino acid, which lowers antioxidant enzyme activity.

Genotoxic alteration following comet assay is a reliable, suitable, sensitive, and extensively used method to measure DNA damage in several cells of terrestrial and aquatic animals [77, 78]. The current study showed a significant rise in % DNA damage in blood cells of BPF-exposed fish under alkaline conditions. DNA damage caused by BPF in the erythrocytes of *L. rohita* has not been previously reported. Kose et al. [79] reported that BPF (1–600 μM for one day) causes DNA damage in RWPE-1 cells. Akram et al. [67] reported the same findings in fish exposed to BPs, describing the genotoxic potential of BPA in bighead carp. Lombó et al. [80] also described that BPA induces DNA damage in zebrafish. This investigation observed a significant rise in OTM in erythrocytes of BPF-exposed fish compared to unexposed fish. The underlying cellular and molecular mechanisms of the genotoxic potential of PBF are still unknown. However, the genotoxicity caused by BPF has been primarily related to oxidative stress by lipid peroxidation [81], which may cause DNA changes [82]. In addition, BPs cause chromosomal aberrations, gene mutations, DNA adduct formation, DNA methylation, and breaking DNA strands [64]. It also changes the DNA repairing mechanism [83]. Furthermore, it may be suggested that DNA damage might also be associated with genetic aberrations caused by BPs, leading to the stimulation of physiologically inactive proteins responsible for breaking nuclear proteins.

The histological approach is a valuable biomarker to estimate the impact of several environmental contaminants on animals [84]. Histopathological biomarkers describe the morphological and physiological status of different organs of animals [85]. Due to their high absorbency and direct contact with the water environment, gills are vital organs for ionic regulation and respiration and a useful biomonitoring tool for evaluating the effects of chemicals in the aquatic atmosphere [86, 87]. In the current investigation, gill tissues of unexposed fish showed normal soft tissues of gill filaments, including primary and secondary gill lamellae (Figure 5(a)). Fish exposed to 600, 1200, and 1800 μg/L of BPF showed various histological alterations such as edema, blood cognition, fusion and degeneration of primary lamellae, bone cell deformities, hyperplasia of epithelial cells, and necrotic cells in the gill tissues (Figures 5(b), 5(c), 5(d)). To date, it is the first report about the histological alterations in the soft tissues of the gills of BPF-exposed fish. Secondary lamellae epithelial protrusion affects oxygen absorption and gas exchange by increasing the distance between blood cells and water. However, fish may adjust by raising their breathing rate [84]. Previously, the same findings on gill changes in fish exposed to BPs were reported [88]. Similar changes were reported in *Catla catla* [89] and *Cyprinus carpio* [47]. Ghafarifarsani et al. [90] observed similar damage in the gills of *Oncorhynchus mykiss* by pesticide exposure. The liver of fish serves as the primary organ responsible for the detoxification of environmental pollutants. Therefore, alterations observed in the liver of aquatic organisms, such as fish, can serve as excellent indicators of aquatic pollution [91]. In this study, no changes were observed in the liver cells of the control fish (Figure 6(a)). Fish exposed to different doses of BPF showed various histological alterations such as degenerative nuclei, necrotic cells, damaged central vein, sinusoidal spaces, and formation of cluster nuclei in liver tissues (Figures 6(b), 6(c), 6(d)). It is the first observation of histological changes in the liver tissues of BPF-exposed fish. Changes in the soft tissues of the liver with sinusoidal spaces and cluster nuclei formation are due to the destruction of structural proteins. The hepatic vein and hepatic artery supply blood to the dorsal aorta. However, the presence of obstruction in the hepatic vein may block the flow of blood. The blockage of the hepatic vein may cause cell damage, and necrosis BPF damages the cell wall, causing necrosis and death of liver cells in exposed fish. The fish may have necrosis due to a lack of new liver cells or a detoxification process that removes harmful substances from the body [92]. Faheem, Jahan, and Lone [89] also observed similar changes in the liver. Abdulla Bin-Dohaish [93] observed the same changes in the soft tissues of the liver of *Oreochromis spilurus* exposed to BPs. The kidneys of fish are the primary osmoregulatory and hematopoietic organs. Histological changes in kidney tissues can serve as an indication of environmental contamination [94, 95]. According to the current study, a photomicrograph of the kidney tissues of *L. rohita* showed a normal arrangement of kidney cells and renal tubules of control fish (Figure 7(a)). Various histological alterations such as melanomacrophage, elongated tubules, tubules with hypertrophied nuclei, sinusoidal spaces, formation of cluster nuclei, and necrotic cells in the kidney of fish exposed to 600, 1200, and 1800 μg/L BPF, respectively (Figures 7(b), 7(c), 7(d)). Previously, no literature was reported about kidney damage by exposure to BPF. Cengiz [94] and Faheem, Jahan, and Lone [89] reported the same findings in fish exposed to BPs. Ghafarifarsani et al. [96] reported that mycotoxins such as aflatoxin B1 and zearalenone also caused histological alterations in *O. mykiss*.

## 5. Conclusion

To our knowledge, this is the first investigation of the toxicological assessment of BPF exposure on freshwater fish rohu *L. rohita*. Specifically, it assesses the impact of BPF on hematobiochemical parameters, DNA damage, and oxidant and antioxidant biomarkers in various organs of exposed fish as well as histological alterations in the liver, gills, and kidneys of exposed fish. According to the current study, BPF causes toxicity by altering hematological and biochemical biomarkers and damaging the soft tissues such as gills, kidneys, and liver. In addition, BPF exposure has significant effects on the immune defense mechanism by increasing oxidative stress content and decreasing the activities of antioxidant enzymes. Nevertheless, DNA damage was also observed in BPF-exposed fish. Since BPF is very toxic to blood biomarkers and different organs of rohu *L. rohita*, preventing BPF from contaminating freshwater bodies is recognized as one of the most important steps to be taken. Immediate actions are needed to prevent future contamination, and discharges of BPF into aquatic ecosystems should be as minimal as feasible.

## Figures and Tables

**Table 1:** The comparison between the biochemical results of T1 (control) and three (T2, T3, and T4) BPF-treated groups.

Parameters	T1 (control)	T2 (600 μg/L)	T3 (1200 μg/L)	T4 (1800 μg/L)
RBC (×10^6^/μL)	1.84 ± 0.06	1.65 ± 0.08⁣^∗^	1.48 ± 0.07⁣^∗∗∗^	1.24 ± 0.10⁣^∗∗∗^
HGB (g/dL)	4.85 ± 0.29	4.12 ± 0.12⁣^∗∗^	3.83 ± 0.18⁣^∗∗∗^	3.71 ± 0.21⁣^∗∗∗^
MCH (pg)	47.31 ± 1.92	43.48 ± 2.08^ns^	39.13 ± 1.69⁣^∗∗^	34.97 ± 1.93⁣^∗∗∗^
MCV (FL)	140.8 ± 1.41	131.8 ± 5.20^ns^	125.0 ± 2.19⁣^∗∗^	121.5 ± 3.34⁣^∗∗^
HCT (%)	15.80 ± 0.69	11.89 ± 1.34⁣^∗∗^	9.47 ± 0.63⁣^∗∗∗^	8.08 ± 0.87⁣^∗∗∗^
MCHC (g/dL)	108.1 ± 2.40	99.10 ± 3.14⁣^∗^	95.43 ± 4.37⁣^∗∗^	89.26 ± 1.83⁣^∗∗∗^
PLT (×10^3^/μL)	195.1 ± 1.17	187.7 ± 2.01^ns^	180.7 ± 5.12⁣^∗∗^	175.0 ± 4.15⁣^∗∗∗^
WBC (×10^3^/μL)	18.89 ± 2.71	27.20 ± 2.89⁣^∗∗^	32.56 ± 1.56⁣^∗∗∗^	36.13 ± 1.59⁣^∗∗∗^
Neutrophils (%)	73.01 ± 1.68	78.20 ± 2.10⁣^∗^	83.53 ± 2.48⁣^∗∗∗^	87.93 ± 1.26⁣^∗∗∗^
Cholesterol (mg/dL)	193.6 ± 2.58	187.8 ± 1.31⁣^∗^	183.0 ± 2.71⁣^∗∗^	177.9 ± 2.35⁣^∗∗∗^
Triglyceride (mg/dL)	212.1 ± 2.39	220.2 ± 2.32^ns^	229.3 ± 4.13⁣^∗∗^	250.4 ± 5.46⁣^∗∗∗^
HDL (mg/dL)	81.05 ± 1.61	76.03 ± 1.62^ns^	71.34 ± 2.67⁣^∗∗^	54.14 ± 3.33⁣^∗∗∗^
LDL (mg/dL)	142.2 ± 2.59	136.9 ± 1.43^ns^	129.2 ± 5.22⁣^∗∗^	122.7 ± 3.45⁣^∗∗∗^
VLDL (mg/dL)	119.9 ± 1.47	115.8 ± 1.52^ns^	107.9 ± 4.66⁣^∗^	100.4 ± 5.36⁣^∗∗∗^
ALT (U/L)	21.07 ± 1.65	27.49 ± 2.18^ns^	33.37 ± 2.76⁣^∗∗^	38.28 ± 3.62⁣^∗∗∗^
AST (U/L)	98.29 ± 2.58	106.9 ± 3.60^ns^	122.0 ± 4.41⁣^∗∗∗^	131.8 ± 6.58⁣^∗∗∗^
Total proteins (mg/dL)	12.65 ± 1.56	9.31 ± 1.11⁣^∗^	8.28 ± 0.87⁣^∗∗^	6.55 ± 0.60⁣^∗∗∗^
Albumin (mg/dL)	4.19 ± 0.31	3.71 ± 0.15^ns^	3.31 ± 0.13⁣^∗∗^	2.77 ± 0.13⁣^∗∗∗^
Globulin (mg/dL)	10.34 ± 0.38	8.92 ± 0.74⁣^∗^	7.44 ± 0.56⁣^∗∗∗^	5.91 ± 0.39⁣^∗∗∗^
Blood glucose (mg/dL)	59.63 ± 5.32	76.11 ± 4.93⁣^∗∗^	94.54 ± 3.57⁣^∗∗∗^	108.4 ± 3.71⁣^∗∗∗^

*Note:* The mean values and standard deviations were calculated using the descriptive statistics tool. The data are represented as mean ± SD. Asterisks represent significant differences between T1 (control) and three (T2, T3, and T4) BPF-treated groups (analyzed by one-way ANOVA by applying Dunnett's post hoc comparison test). *n* = 3 for each experimental group.

Abbreviation: ns = nonsignificant.

⁣^∗^*p* < 0.05.

⁣^∗∗^*p* < 0.01.

⁣^∗∗∗^*p* < 0.001.

## Data Availability

All data and materials are available upon request.
